# Diethyl 1-acetyl-4′-(4-chloro­phen­yl)-5′-(4-nitro­phen­yl)-2-oxospiro­[indoline-3,3′-pyrrolidine]-2′,2′-dicarboxyl­ate

**DOI:** 10.1107/S160053680903551X

**Published:** 2009-09-09

**Authors:** Long He

**Affiliations:** a College of Chemistry and Chemical Engineering, China West Normal University, Nanchong 637002, People’s Republic of China

## Abstract

In the title compound, C_31_H_28_ClN_3_O_8_, the pyrrolidine ring exhibits an envelope conformation, with the spiro C atom located at the flap position. A spiro junction links the oxindole ring system and the pyrrolidine ring. The planar oxindole ring system is twisted with respect to the nitro­benzene and chloro­benzene rings, with dihedral angles of 62.34 (11) and 75.93 (9)°, respectively. In the crystal, a weak C—H⋯O interaction links the molecules into chains and two intramolecular C—H⋯O close contacts are seen.

## Related literature

For general background to the spiro­oxindole–pyrrolidine ring system, see: Garnick & Lequesne (1978 ▶); Jossang *et al.* (1991 ▶). For the biological activity of pyrrolidine-containing compounds and their use in catalysis, see: Grigg (1995 ▶); Kravchenko *et al.* (2005 ▶); Witherup *et al.* (1995 ▶). For the biological activity of oxindole derivatives, see: Glover *et al.* (1998 ▶); Bhattacharya *et al.* (1982 ▶).
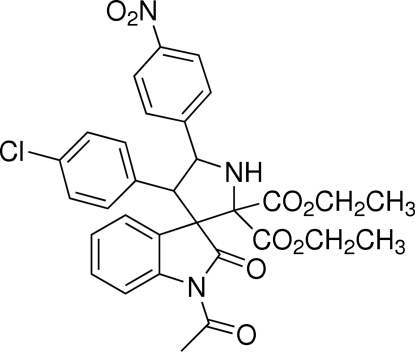


## Experimental

### 

#### Crystal data

C_31_H_28_ClN_3_O_8_
*M* *_r_* = 606.01
Orthorhombic, 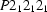

*a* = 9.780 (2) Å
*b* = 14.859 (3) Å
*c* = 20.466 (5) Å
*V* = 2974.1 (11) Å^3^
*Z* = 4
Cu *K*α radiation
μ = 1.61 mm^−1^
*T* = 293 K
0.40 × 0.38 × 0.32 mm

#### Data collection

Oxford Diffraction Gemini S Ultra diffractometer
Absorption correction: multi-scan (*CrysAlis Pro*; Oxford Diffraction, 2009 ▶) *T*_min_ = 0.565, *T*_max_ = 0.626
27366 measured reflections
4732 independent reflections
4456 reflections with *I* > 2σ(*I*)
*R*_int_ = 0.034

#### Refinement

*R*[*F* ^2^ > 2σ(*F* ^2^)] = 0.033
*wR*(*F* ^2^) = 0.066
*S* = 1.00
4732 reflections
403 parameters
H atoms treated by a mixture of independent and constrained refinement
Δρ_max_ = 0.12 e Å^−3^
Δρ_min_ = −0.15 e Å^−3^
Absolute structure: Flack (1983 ▶), with 2017 Friedel pairs
Flack parameter: 0.020 (15)

### 

Data collection: *CrysAlis Pro* (Oxford Diffraction, 2009 ▶); cell refinement: *CrysAlis Pro*; data reduction: *CrysAlis Pro* ; program(s) used to solve structure: *SHELXS97* (Sheldrick, 2008 ▶); program(s) used to refine structure: *SHELXL97* (Sheldrick, 2008 ▶); molecular graphics: *ORTEP-3 for Windows* (Farrugia, 1997 ▶); software used to prepare material for publication: *WinGX* (Farrugia, 1999 ▶).

## Supplementary Material

Crystal structure: contains datablocks global, I. DOI: 10.1107/S160053680903551X/xu2602sup1.cif

Structure factors: contains datablocks I. DOI: 10.1107/S160053680903551X/xu2602Isup2.hkl

Additional supplementary materials:  crystallographic information; 3D view; checkCIF report

## Figures and Tables

**Table 1:** Hydrogen-bond geometry (Å, °)

*D*—H⋯*A*	*D*—H	H⋯*A*	*D*⋯*A*	*D*—H⋯*A*
C10—H10*C*⋯O8^i^	0.96	2.51	3.387 (4)	152
C11—H11⋯O3	0.98	2.54	3.200 (3)	125
C21—H21⋯O3	0.93	2.44	3.181 (3)	136

Symmetry code: (i) 
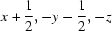
.

**Fig. 1.**
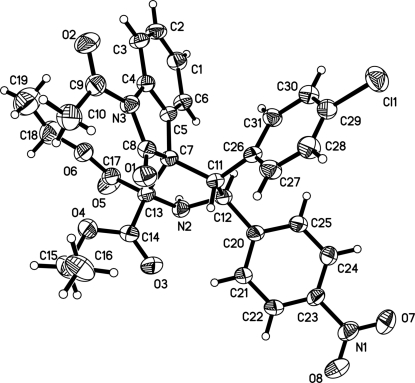
The molecular structure of (I) with 30% probability displacement ellipsoids (arbitrary spheres for H atoms). The minor disordered component is omitted for clarity.

## supplementary crystallographic information

### Comment

The spirooxindole-pyrrolidine unit is a privileged heterocyclic motif that forms the core of a large family of alkaloid natural products with strong bioactivity profiles and interesting structural properties such as alstonia muelleriana and horsfiline (Garnick & Lequesne, 1978; Jossang *et al.*, 1991). Pyrrolidine-containing compounds are of significant importance because of their biological activities and widespread employment in catalysis (Grigg *et al.*, 1995; Witherup *et al.*, 1995; Kravchenko *et al.*, 2005). Oxindole derivatives are of importance in the total synthesis of indole and oxindole alkaloids such as potent inhibitors of monoamine oxidase (MAO) in human urine and rat tissues (Glover *et al.*, 1998) and atrial natriuretic peptide-stimulated guanylate cyclase and a potent antagonist of *in vitro* receptor binding by atrial natriuretic peptide besides possessing a wide range of central nervous system activities (Bhattacharya *et al.*, 1982). We report herein the crystal structure of the title compound.

The molecular structure of (I) is shown in Fig. 1. In the molecule, the pyrrolidine ring exhibits an envelope conformation. The oxyindole ring forms dihedral angles of 62.34 (11)°, 75.93 (9)° with nitrophenyl and chlorophenyl rings, respectively. The crystal packing is stabilized by C—H···O hydrogen bonding (Table 1).

### Experimental

1-acetyl-3-(4-chlorobenzylidene)indolin-2-one (0.09 g, 0.3 mmol), diethyl 2-aminomalonate (0.035 g, 0.2 mmol) and 4-nitrobenzaldehyde (0.036 g, 0.24 mmol) were dissolved in dichloromethane (2 ml). To the stirred mixture, acetic acid (0.006 g, 0.1 mmol) was added. After the mixture had been stirred at 273 K for 24 h, the reaction was quenched with a saturated solution of sodium bicarbonate (5 ml). The mixture was extracted with diethyl ether, removal of solvent under reduced pressure, the residue was purified through column chromatography on silica gel to give target compound. Colourless single crystals suitable for X-ray diffraction were obtained by recrystallization from ethanol.

### Refinement

One ethyl group is disordered over two sites, occupancies were refined to 0.663 (5):0.337 (5). Imino H atoms were located in a difference Fourier map and were refined isotropically. The carbon-bound H atoms were placed in calculated positions, with C—H = 0.93–0.98 Å, and refined using a riding model, *U*_iso_(H) = 1.2*U*_eq_(C).

### Crystal data

**Table:**

C_31_H_28_ClN_3_O_8_	*F*(000) = 1264
*M**_r_* = 606.01	*D*_x_ = 1.353 Mg m^−^^3^
Orthorhombic, *P*2_1_2_1_2_1_	Cu *K*α radiation, λ = 1.54184 Å
Hall symbol: P 2ac 2ab	Cell parameters from 19085 reflections
*a* = 9.780 (2) Å	θ = 2.2–62.6°
*b* = 14.859 (3) Å	µ = 1.61 mm^−^^1^
*c* = 20.466 (5) Å	*T* = 293 K
*V* = 2974.1 (11) Å^3^	Block, colourless
*Z* = 4	0.40 × 0.38 × 0.32 mm

### Data collection

**Table:**

Oxford Diffraction Gemini S Ultra diffractometer	4732 independent reflections
Radiation source: Enhance Ultra (Cu) X-ray Source	4456 reflections with *I* > 2σ(*I*)
mirror	*R*_int_ = 0.034
ω scans	θ_max_ = 62.7°, θ_min_ = 3.7°
Absorption correction: multi-scan (*CrysAlis PRO*; Oxford Diffraction, 2009)	*h* = −11→10
*T*_min_ = 0.565, *T*_max_ = 0.626	*k* = −16→16
27366 measured reflections	*l* = −23→23

### Refinement

**Table:**

Refinement on *F*^2^	Secondary atom site location: difference Fourier map
Least-squares matrix: full	Hydrogen site location: inferred from neighbouring sites
*R*[*F*^2^ > 2σ(*F*^2^)] = 0.033	H atoms treated by a mixture of independent and constrained refinement
*wR*(*F*^2^) = 0.066	*w* = 1/[σ^2^(*F*_o_^2^) + (0.01*P*)^2^ + 1.12*P*] where *P* = (*F*_o_^2^ + 2*F*_c_^2^)/3
*S* = 1.00	(Δ/σ)_max_ = 0.001
4732 reflections	Δρ_max_ = 0.12 e Å^−^^3^
403 parameters	Δρ_min_ = −0.15 e Å^−^^3^
0 restraints	Absolute structure: Flack (1983), with 2017 Friedel pairs
Primary atom site location: structure-invariant direct methods	Flack parameter: 0.020 (15)

### Special details

**Table:**

Geometry. All esds (except the esd in the dihedral angle between two l.s. planes) are estimated using the full covariance matrix. The cell esds are taken into account individually in the estimation of esds in distances, angles and torsion angles; correlations between esds in cell parameters are only used when they are defined by crystal symmetry. An approximate (isotropic) treatment of cell esds is used for estimating esds involving l.s. planes.
Refinement. Refinement of *F*^2^ against ALL reflections. The weighted *R*-factor *wR* and goodness of fit *S* are based on *F*^2^, conventional *R*-factors *R* are based on *F*, with *F* set to zero for negative *F*^2^. The threshold expression of *F*^2^ > σ(*F*^2^) is used only for calculating *R*-factors(gt) *etc*. and is not relevant to the choice of reflections for refinement. *R*-factors based on *F*^2^ are statistically about twice as large as those based on *F*, and *R*- factors based on ALL data will be even larger.

### Fractional atomic coordinates and isotropic or equivalent isotropic displacement parameters (Å^2^)

**Table:**

	*x*	*y*	*z*	*U*_iso_*/*U*_eq_	Occ. (<1)
Cl1	1.09946 (7)	0.04005 (5)	−0.15933 (4)	0.0854 (2)	
O1	0.64684 (17)	−0.06223 (10)	0.10179 (8)	0.0676 (4)	
O2	0.6735 (2)	0.19830 (13)	0.17061 (9)	0.0952 (6)	
O3	0.4061 (2)	−0.20875 (10)	0.04919 (9)	0.0884 (6)	
O4	0.36139 (19)	−0.12113 (11)	0.13466 (8)	0.0711 (5)	
O5	0.13360 (19)	0.01337 (13)	0.03482 (10)	0.0905 (6)	
O6	0.28979 (16)	0.05350 (10)	0.10874 (8)	0.0645 (4)	
O7	0.5892 (3)	−0.37919 (15)	−0.29155 (11)	0.1140 (8)	
O8	0.4917 (3)	−0.45986 (14)	−0.22027 (12)	0.1298 (10)	
N1	0.5298 (3)	−0.38745 (15)	−0.24018 (12)	0.0773 (6)	
N2	0.3198 (2)	−0.07998 (13)	−0.03716 (9)	0.0550 (5)	
H4	0.252 (2)	−0.0467 (15)	−0.0536 (11)	0.057 (7)*	
N3	0.60487 (19)	0.09166 (11)	0.09909 (8)	0.0524 (4)	
C1	0.4012 (3)	0.23572 (15)	−0.04607 (12)	0.0635 (6)	
H1	0.3536	0.2652	−0.0791	0.076*	
C2	0.4675 (3)	0.28445 (15)	0.00113 (13)	0.0653 (6)	
H2	0.4643	0.3470	−0.0006	0.078*	
C3	0.5388 (3)	0.24359 (14)	0.05103 (12)	0.0618 (6)	
H3	0.5826	0.2774	0.0831	0.074*	
C4	0.5428 (2)	0.15029 (13)	0.05182 (10)	0.0500 (5)	
C5	0.4780 (2)	0.09924 (13)	0.00393 (10)	0.0465 (5)	
C6	0.4053 (2)	0.14167 (14)	−0.04455 (11)	0.0554 (5)	
H6	0.3593	0.1082	−0.0760	0.067*	
C7	0.4978 (2)	−0.00011 (13)	0.01833 (10)	0.0463 (5)	
C8	0.5901 (2)	0.00172 (14)	0.07806 (10)	0.0517 (5)	
C9	0.6535 (3)	0.11918 (18)	0.16049 (12)	0.0661 (6)	
C10	0.6741 (4)	0.0499 (2)	0.21150 (12)	0.0956 (9)	
H10A	0.5943	0.0125	0.2142	0.143*	
H10B	0.6893	0.0787	0.2529	0.143*	
H10C	0.7520	0.0137	0.2005	0.143*	
C11	0.5600 (2)	−0.05979 (13)	−0.03652 (10)	0.0475 (5)	
H11	0.5788	−0.1183	−0.0163	0.057*	
C12	0.4372 (2)	−0.07482 (13)	−0.08216 (10)	0.0505 (5)	
H12	0.4265	−0.0221	−0.1105	0.061*	
C13	0.3575 (2)	−0.05148 (13)	0.02874 (10)	0.0501 (5)	
C14	0.3776 (2)	−0.13656 (15)	0.07181 (12)	0.0601 (6)	
C15	0.3784 (3)	−0.19894 (19)	0.17846 (14)	0.0859 (8)	
H15A	0.3345	−0.2511	0.1591	0.103*	
H15B	0.3334	−0.1864	0.2197	0.103*	
C16	0.5243 (4)	−0.2199 (2)	0.19082 (17)	0.1080 (11)	
H16A	0.5308	−0.2698	0.2205	0.162*	
H16B	0.5684	−0.1683	0.2097	0.162*	
H16C	0.5683	−0.2351	0.1504	0.162*	
C17	0.2462 (3)	0.00796 (15)	0.05735 (12)	0.0580 (6)	
C18	0.1908 (18)	0.1190 (11)	0.1331 (5)	0.078 (3)	0.663 (5)
H18A	0.1077	0.0897	0.1479	0.093*	0.663 (5)
H18B	0.1679	0.1630	0.0998	0.093*	0.663 (5)
C19	0.2687 (5)	0.1632 (4)	0.1910 (3)	0.1042 (18)	0.663 (5)
H19A	0.2114	0.2078	0.2112	0.156*	0.663 (5)
H19B	0.3506	0.1912	0.1751	0.156*	0.663 (5)
H19C	0.2920	0.1180	0.2226	0.156*	0.663 (5)
C18B	0.201 (4)	0.109 (2)	0.1530 (12)	0.078 (3)	0.337 (5)
H18C	0.1051	0.0958	0.1446	0.093*	0.337 (5)
H18D	0.2201	0.0945	0.1983	0.093*	0.337 (5)
C19B	0.2277 (11)	0.2020 (8)	0.1410 (6)	0.1042 (18)	0.337 (5)
H19D	0.1689	0.2383	0.1677	0.156*	0.337 (5)
H19E	0.2111	0.2152	0.0957	0.156*	0.337 (5)
H19F	0.3214	0.2150	0.1513	0.156*	0.337 (5)
C20	0.4548 (2)	−0.15803 (14)	−0.12431 (10)	0.0520 (5)	
C21	0.4107 (3)	−0.24182 (15)	−0.10433 (13)	0.0880 (10)	
H21	0.3640	−0.2477	−0.0650	0.106*	
C22	0.4351 (4)	−0.31696 (17)	−0.14218 (13)	0.0920 (10)	
H22	0.4056	−0.3734	−0.1285	0.110*	
C23	0.5028 (2)	−0.30745 (15)	−0.19981 (11)	0.0591 (6)	
C24	0.5454 (3)	−0.22572 (15)	−0.22211 (11)	0.0637 (6)	
H24	0.5899	−0.2204	−0.2621	0.076*	
C25	0.5206 (3)	−0.15126 (15)	−0.18380 (11)	0.0612 (6)	
H25	0.5489	−0.0950	−0.1984	0.073*	
C26	0.6926 (2)	−0.02983 (13)	−0.06711 (10)	0.0482 (5)	
C27	0.8076 (2)	−0.08192 (15)	−0.05669 (12)	0.0619 (6)	
H27	0.8008	−0.1323	−0.0300	0.074*	
C28	0.9330 (2)	−0.06145 (17)	−0.08482 (12)	0.0688 (7)	
H28	1.0089	−0.0978	−0.0775	0.083*	
C29	0.9422 (2)	0.01330 (16)	−0.12345 (11)	0.0611 (6)	
C30	0.8322 (2)	0.06705 (15)	−0.13547 (11)	0.0622 (6)	
H30	0.8408	0.1175	−0.1621	0.075*	
C31	0.7064 (2)	0.04538 (15)	−0.10724 (11)	0.0583 (5)	
H31	0.6307	0.0816	−0.1153	0.070*	

### Atomic displacement parameters (Å^2^)

**Table:**

	*U*^11^	*U*^22^	*U*^33^	*U*^12^	*U*^13^	*U*^23^
Cl1	0.0629 (4)	0.1033 (5)	0.0900 (5)	−0.0142 (4)	0.0124 (3)	0.0004 (4)
O1	0.0756 (11)	0.0570 (9)	0.0701 (10)	0.0076 (8)	−0.0155 (8)	0.0078 (8)
O2	0.1405 (18)	0.0780 (13)	0.0672 (11)	−0.0327 (13)	−0.0184 (12)	−0.0167 (10)
O3	0.1411 (18)	0.0437 (9)	0.0805 (12)	0.0019 (10)	0.0153 (13)	0.0014 (9)
O4	0.0926 (13)	0.0613 (10)	0.0595 (10)	0.0072 (9)	0.0060 (9)	0.0083 (8)
O5	0.0607 (11)	0.0989 (14)	0.1119 (15)	0.0141 (10)	−0.0127 (11)	−0.0356 (12)
O6	0.0650 (9)	0.0594 (9)	0.0692 (10)	0.0006 (8)	0.0083 (8)	−0.0157 (8)
O7	0.154 (2)	0.0987 (15)	0.0888 (15)	−0.0116 (15)	0.0374 (16)	−0.0396 (12)
O8	0.195 (3)	0.0629 (13)	0.1315 (19)	−0.0220 (16)	0.0478 (19)	−0.0379 (13)
N1	0.0878 (16)	0.0643 (14)	0.0798 (16)	−0.0093 (12)	0.0046 (13)	−0.0251 (12)
N2	0.0546 (11)	0.0537 (11)	0.0566 (11)	−0.0051 (9)	−0.0018 (9)	−0.0087 (9)
N3	0.0583 (11)	0.0502 (10)	0.0487 (10)	−0.0053 (8)	−0.0071 (9)	−0.0043 (8)
C1	0.0697 (15)	0.0501 (13)	0.0708 (15)	0.0102 (12)	0.0029 (13)	0.0104 (11)
C2	0.0719 (16)	0.0399 (12)	0.0840 (17)	−0.0005 (11)	0.0056 (14)	0.0018 (12)
C3	0.0664 (14)	0.0449 (12)	0.0739 (15)	−0.0098 (11)	0.0039 (13)	−0.0123 (11)
C4	0.0516 (11)	0.0450 (11)	0.0533 (12)	−0.0037 (9)	0.0016 (10)	−0.0026 (10)
C5	0.0514 (12)	0.0369 (10)	0.0510 (12)	0.0004 (9)	0.0002 (9)	−0.0026 (9)
C6	0.0590 (13)	0.0463 (12)	0.0610 (13)	0.0031 (10)	−0.0055 (11)	−0.0019 (10)
C7	0.0525 (12)	0.0377 (10)	0.0487 (11)	0.0001 (9)	−0.0032 (9)	−0.0006 (9)
C8	0.0529 (12)	0.0497 (12)	0.0523 (12)	−0.0009 (10)	0.0001 (10)	0.0019 (10)
C9	0.0693 (15)	0.0755 (17)	0.0534 (13)	−0.0155 (13)	−0.0037 (12)	−0.0043 (13)
C10	0.132 (3)	0.098 (2)	0.0573 (15)	−0.014 (2)	−0.0244 (17)	0.0039 (15)
C11	0.0546 (12)	0.0357 (10)	0.0521 (11)	−0.0015 (9)	−0.0004 (10)	−0.0010 (9)
C12	0.0595 (13)	0.0397 (11)	0.0523 (12)	−0.0044 (9)	0.0005 (10)	−0.0022 (9)
C13	0.0540 (12)	0.0412 (11)	0.0552 (12)	−0.0020 (9)	0.0000 (10)	−0.0038 (9)
C14	0.0651 (14)	0.0495 (13)	0.0658 (15)	−0.0077 (11)	0.0064 (12)	0.0007 (11)
C15	0.105 (2)	0.0771 (18)	0.0751 (17)	0.0016 (17)	0.0107 (17)	0.0251 (15)
C16	0.112 (3)	0.100 (2)	0.112 (3)	0.006 (2)	−0.011 (2)	0.048 (2)
C17	0.0604 (14)	0.0491 (12)	0.0645 (14)	−0.0033 (10)	0.0052 (12)	−0.0050 (11)
C18	0.078 (3)	0.076 (4)	0.080 (7)	0.010 (3)	0.007 (6)	−0.031 (5)
C19	0.092 (3)	0.116 (4)	0.105 (4)	−0.007 (3)	0.015 (3)	−0.060 (3)
C18B	0.078 (3)	0.076 (4)	0.080 (7)	0.010 (3)	0.007 (6)	−0.031 (5)
C19B	0.092 (3)	0.116 (4)	0.105 (4)	−0.007 (3)	0.015 (3)	−0.060 (3)
C20	0.0603 (13)	0.0450 (11)	0.0507 (12)	−0.0084 (10)	−0.0007 (10)	−0.0051 (9)
C21	0.138 (3)	0.0526 (14)	0.0734 (16)	−0.0306 (16)	0.0429 (18)	−0.0163 (13)
C22	0.141 (3)	0.0499 (14)	0.0848 (19)	−0.0320 (16)	0.0378 (19)	−0.0142 (13)
C23	0.0673 (15)	0.0525 (13)	0.0576 (13)	−0.0043 (11)	0.0008 (11)	−0.0163 (11)
C24	0.0789 (16)	0.0616 (14)	0.0506 (12)	−0.0091 (13)	0.0038 (12)	−0.0063 (11)
C25	0.0818 (17)	0.0478 (12)	0.0540 (13)	−0.0077 (12)	0.0038 (12)	0.0027 (10)
C26	0.0533 (11)	0.0392 (11)	0.0522 (12)	−0.0026 (9)	−0.0021 (10)	−0.0053 (9)
C27	0.0607 (14)	0.0519 (13)	0.0731 (15)	0.0013 (11)	0.0011 (12)	0.0100 (11)
C28	0.0524 (14)	0.0690 (16)	0.0849 (18)	0.0069 (12)	0.0008 (12)	0.0059 (14)
C29	0.0580 (14)	0.0645 (14)	0.0608 (13)	−0.0121 (11)	0.0028 (11)	−0.0111 (11)
C30	0.0674 (15)	0.0516 (13)	0.0674 (15)	−0.0039 (11)	0.0043 (12)	0.0038 (11)
C31	0.0620 (13)	0.0495 (12)	0.0635 (13)	0.0034 (11)	0.0037 (11)	0.0053 (11)

### Geometric parameters (Å, °)

**Table:**

Cl1—C29	1.750 (2)	C12—H12	0.9800
O1—C8	1.203 (2)	C13—C17	1.519 (3)
O2—C9	1.210 (3)	C13—C14	1.554 (3)
O3—C14	1.201 (3)	C15—C16	1.482 (4)
O4—C14	1.316 (3)	C15—H15A	0.9700
O4—C15	1.472 (3)	C15—H15B	0.9700
O5—C17	1.197 (3)	C16—H16A	0.9600
O6—C17	1.321 (3)	C16—H16B	0.9600
O6—C18	1.460 (16)	C16—H16C	0.9600
O6—C18B	1.50 (3)	C18—C19	1.555 (15)
O7—N1	1.208 (3)	C18—H18A	0.9700
O8—N1	1.209 (3)	C18—H18B	0.9700
N1—C23	1.471 (3)	C19—H19A	0.9600
N2—C13	1.461 (3)	C19—H19B	0.9600
N2—C12	1.474 (3)	C19—H19C	0.9600
N2—H4	0.90 (2)	C18B—C19B	1.43 (4)
N3—C9	1.404 (3)	C18B—H18C	0.9700
N3—C8	1.411 (3)	C18B—H18D	0.9700
N3—C4	1.437 (3)	C19B—H19D	0.9600
C1—C2	1.370 (3)	C19B—H19E	0.9600
C1—C6	1.398 (3)	C19B—H19F	0.9600
C1—H1	0.9300	C20—C21	1.380 (3)
C2—C3	1.377 (3)	C20—C25	1.381 (3)
C2—H2	0.9300	C21—C22	1.380 (3)
C3—C4	1.387 (3)	C21—H21	0.9300
C3—H3	0.9300	C22—C23	1.360 (3)
C4—C5	1.392 (3)	C22—H22	0.9300
C5—C6	1.374 (3)	C23—C24	1.363 (3)
C5—C7	1.518 (3)	C24—C25	1.378 (3)
C6—H6	0.9300	C24—H24	0.9300
C7—C8	1.520 (3)	C25—H25	0.9300
C7—C11	1.554 (3)	C26—C27	1.382 (3)
C7—C13	1.585 (3)	C26—C31	1.393 (3)
C9—C10	1.479 (4)	C27—C28	1.388 (3)
C10—H10A	0.9600	C27—H27	0.9300
C10—H10B	0.9600	C28—C29	1.366 (3)
C10—H10C	0.9600	C28—H28	0.9300
C11—C26	1.508 (3)	C29—C30	1.362 (3)
C11—C12	1.537 (3)	C30—C31	1.398 (3)
C11—H11	0.9800	C30—H30	0.9300
C12—C20	1.517 (3)	C31—H31	0.9300
			
C14—O4—C15	116.40 (19)	O4—C15—C16	112.2 (2)
C17—O6—C18	113.5 (6)	O4—C15—H15A	109.2
C17—O6—C18B	125.0 (13)	C16—C15—H15A	109.2
C18—O6—C18B	17.2 (12)	O4—C15—H15B	109.2
O7—N1—O8	122.1 (2)	C16—C15—H15B	109.2
O7—N1—C23	119.5 (2)	H15A—C15—H15B	107.9
O8—N1—C23	118.3 (2)	C15—C16—H16A	109.5
C13—N2—C12	111.43 (17)	C15—C16—H16B	109.5
C13—N2—H4	112.0 (15)	H16A—C16—H16B	109.5
C12—N2—H4	108.5 (15)	C15—C16—H16C	109.5
C9—N3—C8	125.68 (18)	H16A—C16—H16C	109.5
C9—N3—C4	124.68 (18)	H16B—C16—H16C	109.5
C8—N3—C4	109.00 (16)	O5—C17—O6	124.7 (2)
C2—C1—C6	119.9 (2)	O5—C17—C13	123.4 (2)
C2—C1—H1	120.0	O6—C17—C13	112.0 (2)
C6—C1—H1	120.0	O6—C18—C19	102.5 (9)
C1—C2—C3	121.9 (2)	O6—C18—H18A	111.3
C1—C2—H2	119.0	C19—C18—H18A	111.3
C3—C2—H2	119.0	O6—C18—H18B	111.3
C2—C3—C4	117.6 (2)	C19—C18—H18B	111.3
C2—C3—H3	121.2	H18A—C18—H18B	109.2
C4—C3—H3	121.2	C19B—C18B—O6	109 (2)
C3—C4—C5	121.6 (2)	C19B—C18B—H18C	110.0
C3—C4—N3	128.8 (2)	O6—C18B—H18C	110.0
C5—C4—N3	109.64 (16)	C19B—C18B—H18D	110.0
C6—C5—C4	119.60 (18)	O6—C18B—H18D	110.0
C6—C5—C7	130.76 (18)	H18C—C18B—H18D	108.4
C4—C5—C7	109.57 (18)	C18B—C19B—H19D	109.5
C5—C6—C1	119.3 (2)	C18B—C19B—H19E	109.5
C5—C6—H6	120.3	H19D—C19B—H19E	109.5
C1—C6—H6	120.3	C18B—C19B—H19F	109.5
C5—C7—C8	102.40 (16)	H19D—C19B—H19F	109.5
C5—C7—C11	117.69 (17)	H19E—C19B—H19F	109.5
C8—C7—C11	111.05 (16)	C21—C20—C25	118.2 (2)
C5—C7—C13	112.56 (17)	C21—C20—C12	122.09 (19)
C8—C7—C13	114.50 (17)	C25—C20—C12	119.66 (19)
C11—C7—C13	99.26 (15)	C22—C21—C20	120.7 (2)
O1—C8—N3	125.27 (19)	C22—C21—H21	119.7
O1—C8—C7	125.77 (19)	C20—C21—H21	119.7
N3—C8—C7	108.86 (17)	C23—C22—C21	119.1 (2)
O2—C9—N3	119.4 (2)	C23—C22—H22	120.4
O2—C9—C10	122.2 (2)	C21—C22—H22	120.4
N3—C9—C10	118.4 (2)	C22—C23—C24	122.1 (2)
C9—C10—H10A	109.5	C22—C23—N1	119.4 (2)
C9—C10—H10B	109.5	C24—C23—N1	118.5 (2)
H10A—C10—H10B	109.5	C23—C24—C25	118.1 (2)
C9—C10—H10C	109.5	C23—C24—H24	120.9
H10A—C10—H10C	109.5	C25—C24—H24	120.9
H10B—C10—H10C	109.5	C24—C25—C20	121.7 (2)
C26—C11—C12	117.55 (17)	C24—C25—H25	119.2
C26—C11—C7	117.90 (16)	C20—C25—H25	119.2
C12—C11—C7	102.50 (16)	C27—C26—C31	117.5 (2)
C26—C11—H11	106.0	C27—C26—C11	118.09 (18)
C12—C11—H11	106.0	C31—C26—C11	124.4 (2)
C7—C11—H11	106.0	C26—C27—C28	122.1 (2)
N2—C12—C20	113.65 (17)	C26—C27—H27	118.9
N2—C12—C11	103.66 (16)	C28—C27—H27	118.9
C20—C12—C11	112.04 (18)	C29—C28—C27	118.5 (2)
N2—C12—H12	109.1	C29—C28—H28	120.8
C20—C12—H12	109.1	C27—C28—H28	120.8
C11—C12—H12	109.1	C30—C29—C28	121.9 (2)
N2—C13—C17	110.12 (18)	C30—C29—Cl1	119.01 (18)
N2—C13—C14	108.65 (17)	C28—C29—Cl1	119.05 (19)
C17—C13—C14	110.18 (18)	C29—C30—C31	119.1 (2)
N2—C13—C7	103.52 (16)	C29—C30—H30	120.5
C17—C13—C7	113.09 (16)	C31—C30—H30	120.5
C14—C13—C7	111.02 (17)	C26—C31—C30	120.9 (2)
O3—C14—O4	124.1 (2)	C26—C31—H31	119.6
O3—C14—C13	122.5 (2)	C30—C31—H31	119.6
O4—C14—C13	113.43 (19)		
			
C6—C1—C2—C3	−0.3 (4)	C8—C7—C13—C14	−38.4 (2)
C1—C2—C3—C4	0.7 (4)	C11—C7—C13—C14	79.90 (19)
C2—C3—C4—C5	0.3 (4)	C15—O4—C14—O3	−0.8 (4)
C2—C3—C4—N3	−177.5 (2)	C15—O4—C14—C13	179.8 (2)
C9—N3—C4—C3	12.0 (4)	N2—C13—C14—O3	24.4 (3)
C8—N3—C4—C3	−176.8 (2)	C17—C13—C14—O3	145.1 (3)
C9—N3—C4—C5	−166.0 (2)	C7—C13—C14—O3	−88.8 (3)
C8—N3—C4—C5	5.3 (2)	N2—C13—C14—O4	−156.19 (19)
C3—C4—C5—C6	−1.6 (3)	C17—C13—C14—O4	−35.5 (3)
N3—C4—C5—C6	176.57 (19)	C7—C13—C14—O4	90.6 (2)
C3—C4—C5—C7	−178.9 (2)	C14—O4—C15—C16	79.3 (3)
N3—C4—C5—C7	−0.8 (2)	C18—O6—C17—O5	−5.4 (7)
C4—C5—C6—C1	1.9 (3)	C18B—O6—C17—O5	9.4 (15)
C7—C5—C6—C1	178.6 (2)	C18—O6—C17—C13	173.4 (7)
C2—C1—C6—C5	−1.0 (4)	C18B—O6—C17—C13	−171.8 (15)
C6—C5—C7—C8	179.5 (2)	N2—C13—C17—O5	15.1 (3)
C4—C5—C7—C8	−3.5 (2)	C14—C13—C17—O5	−104.8 (3)
C6—C5—C7—C11	57.4 (3)	C7—C13—C17—O5	130.3 (2)
C4—C5—C7—C11	−125.59 (19)	N2—C13—C17—O6	−163.76 (18)
C6—C5—C7—C13	−57.1 (3)	C14—C13—C17—O6	76.4 (2)
C4—C5—C7—C13	119.90 (19)	C7—C13—C17—O6	−48.5 (2)
C9—N3—C8—O1	−19.7 (4)	C17—O6—C18—C19	−178.6 (6)
C4—N3—C8—O1	169.1 (2)	C18B—O6—C18—C19	46 (7)
C9—N3—C8—C7	163.62 (19)	C17—O6—C18B—C19B	−108 (2)
C4—N3—C8—C7	−7.5 (2)	C18—O6—C18B—C19B	−55 (6)
C5—C7—C8—O1	−169.9 (2)	N2—C12—C20—C21	−27.4 (3)
C11—C7—C8—O1	−43.5 (3)	C11—C12—C20—C21	89.7 (3)
C13—C7—C8—O1	67.9 (3)	N2—C12—C20—C25	154.6 (2)
C5—C7—C8—N3	6.7 (2)	C11—C12—C20—C25	−88.3 (2)
C11—C7—C8—N3	133.17 (18)	C25—C20—C21—C22	1.7 (4)
C13—C7—C8—N3	−115.43 (19)	C12—C20—C21—C22	−176.3 (3)
C8—N3—C9—O2	173.1 (3)	C20—C21—C22—C23	−0.3 (5)
C4—N3—C9—O2	−17.0 (4)	C21—C22—C23—C24	−1.2 (5)
C8—N3—C9—C10	−8.8 (4)	C21—C22—C23—N1	179.6 (3)
C4—N3—C9—C10	161.0 (2)	O7—N1—C23—C22	179.6 (3)
C5—C7—C11—C26	52.6 (3)	O8—N1—C23—C22	−0.7 (4)
C8—C7—C11—C26	−64.9 (2)	O7—N1—C23—C24	0.4 (4)
C13—C7—C11—C26	174.27 (17)	O8—N1—C23—C24	−179.9 (3)
C5—C7—C11—C12	−78.2 (2)	C22—C23—C24—C25	1.3 (4)
C8—C7—C11—C12	164.33 (16)	N1—C23—C24—C25	−179.5 (2)
C13—C7—C11—C12	43.47 (17)	C23—C24—C25—C20	0.1 (4)
C13—N2—C12—C20	133.41 (19)	C21—C20—C25—C24	−1.6 (4)
C13—N2—C12—C11	11.5 (2)	C12—C20—C25—C24	176.5 (2)
C26—C11—C12—N2	−165.99 (17)	C12—C11—C26—C27	−123.4 (2)
C7—C11—C12—N2	−34.98 (18)	C7—C11—C26—C27	113.1 (2)
C26—C11—C12—C20	71.1 (2)	C12—C11—C26—C31	54.3 (3)
C7—C11—C12—C20	−157.92 (16)	C7—C11—C26—C31	−69.3 (3)
C12—N2—C13—C17	137.36 (18)	C31—C26—C27—C28	−0.2 (3)
C12—N2—C13—C14	−101.9 (2)	C11—C26—C27—C28	177.6 (2)
C12—N2—C13—C7	16.2 (2)	C26—C27—C28—C29	0.7 (4)
C5—C7—C13—N2	88.8 (2)	C27—C28—C29—C30	−0.7 (4)
C8—C7—C13—N2	−154.82 (17)	C27—C28—C29—Cl1	−179.96 (19)
C11—C7—C13—N2	−36.52 (19)	C28—C29—C30—C31	0.3 (4)
C5—C7—C13—C17	−30.4 (2)	Cl1—C29—C30—C31	179.52 (18)
C8—C7—C13—C17	86.0 (2)	C27—C26—C31—C30	−0.3 (3)
C11—C7—C13—C17	−155.66 (17)	C11—C26—C31—C30	−177.9 (2)
C5—C7—C13—C14	−154.82 (17)	C29—C30—C31—C26	0.2 (3)

### Hydrogen-bond geometry (Å, °)

**Table:**

*D*—H···*A*	*D*—H	H···*A*	*D*···*A*	*D*—H···*A*
C10—H10C···O8^i^	0.96	2.51	3.387 (4)	152
C11—H11···O3	0.98	2.54	3.200 (3)	125
C21—H21···O3	0.93	2.44	3.181 (3)	136

Symmetry codes: (i) *x*+1/2, −*y*−1/2, −*z*.
